# Clinical characteristics and outcomes in childhood-onset hypertrophic cardiomyopathy

**DOI:** 10.1093/eurheartj/ehab148

**Published:** 2021-03-26

**Authors:** Nicholas A Marston, Larry Han, Iacopo Olivotto, Sharlene M Day, Euan A Ashley, Michelle Michels, Alexandre C Pereira, Jodie Ingles, Christopher Semsarian, Daniel Jacoby, Steven D Colan, Joseph W Rossano, Samuel G Wittekind, James S Ware, Sara Saberi, Adam S Helms, Carolyn Y Ho

**Affiliations:** Division of Cardiology, Department of Medicine, Brigham and Women’s Hospital, Harvard Medical School, 75 Francis Street, Boston, MA 02115, USA; TIMI Study Group, Boston, MA, USA; Department of Biostatistics, Harvard T.H. Chan School of Public Health, Boston, MA, USA; Cardiomyopathy Unit, Careggi University Hospital, Florence, Italy; Department of Internal Medicine, University of Pennsylvania, Philadelphia, PA, USA; Stanford Center for Inherited Heart Disease, Stanford, CA, USA; Department of Cardiology, Thoraxcenter, Erasmus MC Rotterdam, The Netherlands; University of Sao Paulo Medical School, Brazil; Department of Cardiology, Royal Prince Alfred Hospital, Agnes Ginges Centre for Molecular Cardiology, at Centenary Institute, The University of Sydney, Australia; Department of Cardiology, Royal Prince Alfred Hospital, Agnes Ginges Centre for Molecular Cardiology, at Centenary Institute, The University of Sydney, Australia; Yale University, New Haven, CT, USA; Boston Children’s Hospital, Harvard Medical School, Boston, MA, USA; Children’s Hospital of Philadelphia, PA, USA; Cincinnati Children's Hospital Medical Center, Heart Institute, Cincinnati, OH, USA; National Heart & Lung Institute & Royal Brompton Cardiovascular Research Centre, Imperial College London, London, England; Department of Internal Medicine-Cardiology, University of Michigan, Ann Arbor, MI, USA; Department of Internal Medicine-Cardiology, University of Michigan, Ann Arbor, MI, USA; Division of Cardiology, Department of Medicine, Brigham and Women’s Hospital, Harvard Medical School, 75 Francis Street, Boston, MA 02115, USA

**Keywords:** Hypertrophic cardiomyopathy, Genetics, Heart failure, Atrial fibrillation, Ventricular arrhythmias

## Abstract

**Aims:**

Childhood-onset hypertrophic cardiomyopathy (HCM) is far less common than adult-onset disease, thus natural history is not well characterized. We aim to describe the characteristics and outcomes of childhood-onset HCM.

**Methods and results:**

We performed an observational cohort study of 7677 HCM patients from the Sarcomeric Human Cardiomyopathy Registry (SHaRe). Hypertrophic cardiomyopathy patients were stratified by age at diagnosis [<1 year (infancy), 1–18 years (childhood), >18 years (adulthood)] and assessed for composite endpoints reflecting heart failure (HF), life-threatening ventricular arrhythmias, atrial fibrillation (AF), and an overall composite that also included stroke and death. Stratifying by age of diagnosis, 184 (2.4%) patients were diagnosed in infancy; 1128 (14.7%) in childhood; and 6365 (82.9%) in adulthood. Childhood-onset HCM patients had an ∼2%/year event rate for the overall composite endpoint, with ventricular arrhythmias representing the most common event in the 1st decade following baseline visit, but HF and AF becoming more common by the end of the 2nd decade. Sarcomeric variants were more common in childhood-onset HCM (63%) and carried a worse prognosis than non-sarcomeric disease, including a greater than two-fold increased risk of HF [HR_adj_ 2.39 (1.36–4.20), *P* = 0.003] and 67% increased risk of the overall composite outcome [HR_adj_ 1.67 (1.16–2.41), *P* = 0.006]. When compared with adult-onset HCM, childhood-onset was 36% more likely to develop life-threatening ventricular arrhythmias [HR_adj_ 1.36 (1.03–1.80)] and twice as likely to require transplant or ventricular assist device [HR_adj_ 1.99 (1.23–3.23)].

**Conclusion:**

Patients with childhood-onset HCM are more likely to have sarcomeric disease, carry a higher risk of life-threatening ventricular arrythmias, and have greater need for advanced HF therapies. These findings provide insight into the natural history of disease and can help inform clinical risk stratification.

## Graphical Abstract

**Figure:**
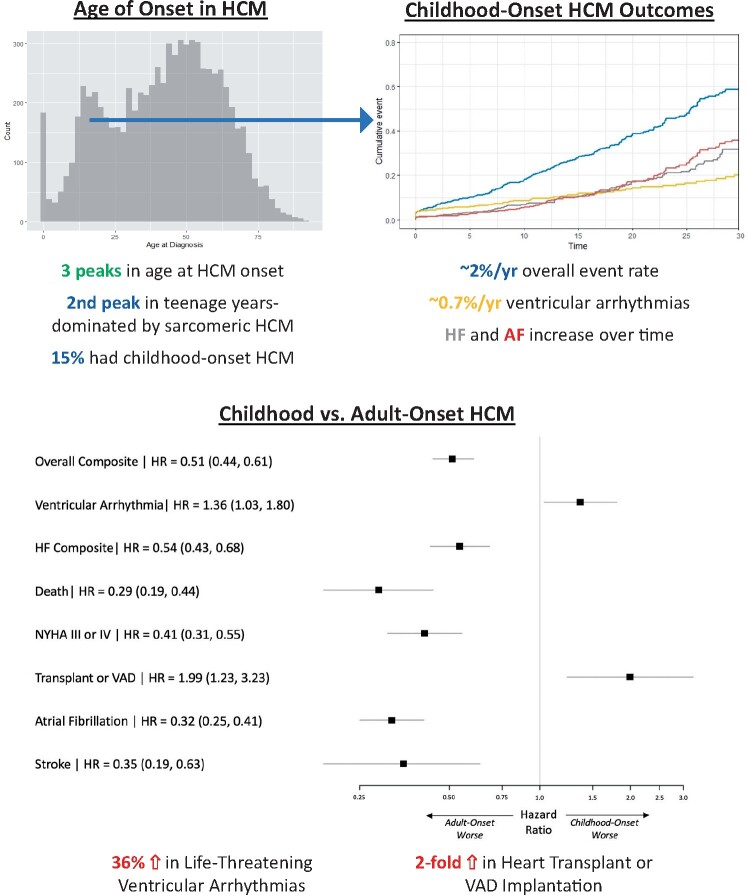
Natural history of sarcomeric HCM may differ depending on age of diagnosis with HCM.

**See page 1997 for the editorial comment on this article (doi: 10.1093/eurheartj/ehab093)**

## Introduction

Hypertrophic cardiomyopathy (HCM) is characterized by unexplained left ventricular hypertrophy (LVH) and often caused by pathogenic variants in genes that encode the sarcomere apparatus. Patients with HCM may experience atrial and ventricular arrhythmias and heart failure (HF); however, disease expression and severity are highly variable. Furthermore, there is marked diversity in the age of diagnosis. The estimated prevalence of HCM is 1:500 in the general adult population.^1^ Although childhood-onset disease is well documented, it is far less common, with an estimated prevalence of 0.24–0.47/100 000.^2^ ^,^ ^3^ Owing to its rarity, the natural history of childhood-onset HCM is not well characterized.

Prevailing beliefs are that HCM typically develops during adolescence and that childhood-onset disease is associated with an increased risk of ventricular arrhythmias, but lower risk of HF and atrial fibrillation (AF). However, data to support these beliefs are limited as prior studies have been small, included a large proportion of infant-onset cardiomyopathy (diagnosed in the 1st year of life), and often included syndromic causes of cardiac hypertrophy, such as Friedreich’s ataxia, Noonan’s syndrome/RASopathies, and inborn errors of metabolism—all of which are distinct disorders from primary HCM.^4–6^

In this study, we aimed to describe the characteristics and outcomes of childhood-onset primary HCM in a large multicentre cohort. This study focuses on patients diagnosed between 1 and 18 years of age, describing adverse cardiac outcomes and risk predictors, determining the impact of pathogenic variation in sarcomeric genes, and characterizing how natural history differs in patients diagnosed with HCM during childhood vs. adulthood.

## Methods

### Study design

The Sarcomeric Human Cardiomyopathy Registry (SHaRe) is an ongoing longitudinal registry study drawing from HCM referral centres across the USA, Brazil, Europe, and Australia. Data captured include historical events prior to SHaRe entry, baseline demographic and clinical data at first visit to a SHaRe site, genetic testing results, serial cardiac imaging results, and longitudinal cardiovascular outcomes. Each site provides updated data on a quarterly basis. A detailed description of SHaRe has previously been published.^7^

### Study population

Patients included in this study carried a site-designated diagnosis of HCM. This was defined as maximal left ventricular wall thickness of at least 13 mm (or body surface area adjusted Z-score >2 for paediatric patients^8^), taking into account non-cardiac findings, family history, and genotype. In addition, all patients were required to have at least one clinic visit at a SHaRe site and at least one echocardiographic assessment. For this analysis, we categorized patients by the following ages at diagnosis: <1 (infant-onset), 1–18 (childhood-onset), >18 (adult-onset) years. For selected sub-analyses, we further stratified patients diagnosed in adulthood by age of diagnosis 19–39 and ≥40 years.

### Genetic testing

Genetic testing was performed by sites as part of research protocols or clinical care. As previously described,^7^ contemporary approaches and American College of Medical Genetics and Genomics (ACMG) guidelines were used to classify sarcomere gene variants as pathogenic, likely pathogenic, of unknown significance, or likely benign/benign^9^ ^,^ ^10^ focusing on the 8 core sarcomeric genes: myosin-binding protein C (*MYBPC3*), myosin heavy chain (*MYH7*), cardiac troponin T (*TNNT2*), cardiac troponin I (*TNNI3*), α-tropomyosin (*TPM1*), myosin essential and regulatory light chains (*MLY2, MYL3*), and actin (*ACTC*). Sarcomeric variants with ambiguous classifications underwent additional systematic review by a subgroup of four investigators (S.M.D., J.I., J.S.W., and C.Y.H.) to adjudicate and standardize classification.^7^ ^,^ ^11^ Patients with at least one pathogenic or likely pathogenic variant in sarcomeric genes were classified as sarcomeric HCM and those with only benign/likely benign variants or no clinically significant sarcomeric variants identified were classified as non-sarcomeric HCM. For analyses comparing sarcomeric and non-sarcomeric HCM, patients who did not undergo genetic testing or who had variants of unknown significance in sarcomeric genes were excluded. Patients with pathogenic variants in non-sarcomeric proteins were excluded entirely from this study [i.e. RASopathies, lysosome-associated membrane protein (*LAMP2*) and α-galactosidase (*GLA*)] as these identify genocopies resulting in LVH and different disease states.

### Endpoints

The endpoints of interest included an HF composite, life-threatening ventricular arrhythmia composite, AF, and an overall cardiac event composite. The HF composite included left ventricular ejection fraction <35%, New York Heart Association (NYHA) functional class III/IV symptoms, cardiac transplantation, or implantation of a left ventricular assist device. The life-threatening ventricular arrhythmia composite included sudden cardiac death, resuscitated cardiac arrest, or appropriate implantable cardioverter-defibrillator therapy. The overall composite included components of the HF composite (excluding left ventricular ejection fraction <35%), components of the ventricular arrhythmia composite, and additionally AF, stroke, and all-cause death.

### Statistical analysis

This analysis included patient data obtained through the 3rd quarter of 2019. All analyses were stratified by age at diagnosis, and time to first event was evaluated from initial visit at a SHaRe site forward. Analyses comparing sarcomeric to non-sarcomeric patients were limited to patients who underwent genetic testing. Continuous variables within each age group are presented as median and interquartile ranges and compared using Kruskal–Wallis test. Categorical variables are compared using a χ^2^ test. Kaplan–Meier analyses were performed using the first occurrence of any component of the composite outcome of interest. Cox-proportional hazards regression model was used to generate hazard ratios (HRs) and 95% confidence intervals (CIs). A multivariable model including risk predictors selected based on biological importance in disease was performed on patients who had complete data available for all relevant variables. All reported *P*-values are two-sided. All analyses were performed using R version 3.5.4 (R Core Team, 2019).

## Results

### Overall cohort

**Figure 1:**
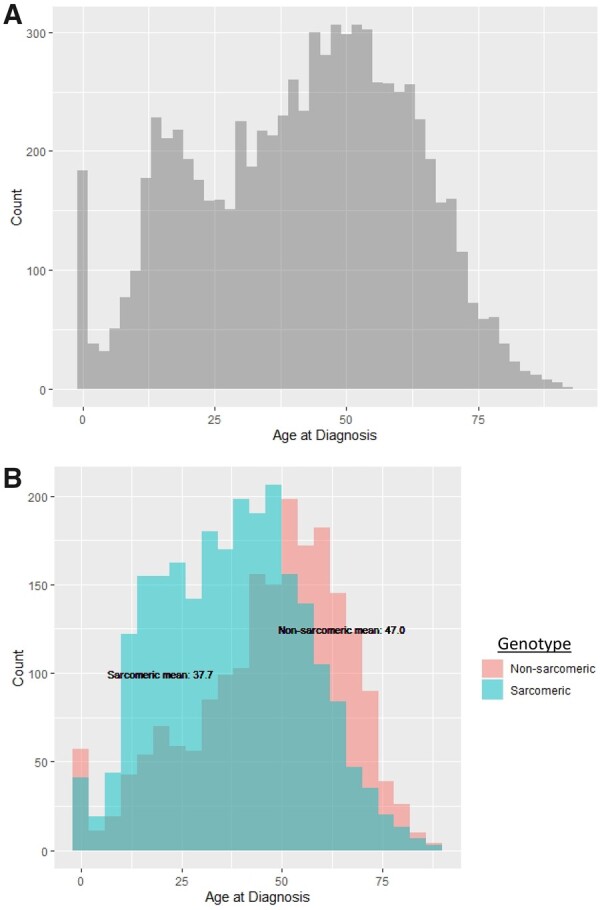
Age at diagnosis with hypertrophic cardiomyopathy. (*A*) Distribution of age at diagnosis in patients with hypertrophic cardiomyopathy (*n* = 7677). There are three peaks when hypertrophic cardiomyopathy is diagnosed throughout the lifespan: infancy (the 1st year of life), adolescence (teenage years), and middle adulthood (∼50 years of age). (*B*) Distribution of age at diagnosis in patients with genetic testing (*n* = 4746), stratified by sarcomeric variant status. Sarcomeric hypertrophic cardiomyopathy (teal) presents 9.3 years earlier than non-sarcomeric hypertrophic cardiomyopathy (pink). Sarcomeric hypertrophic cardiomyopathy is more common prior to age 50, and non-sarcomeric hypertrophic cardiomyopathy is more common thereafter.

There were 7677 HCM patients included in this cohort. *Figure 1A* shows the distribution of age at HCM diagnosis. Across the lifespan, there appear to be three predominant peaks when HCM is diagnosed: the first is within the 1st year of life (infancy), the second is in the teenage to early adult years, and the third is in mid-adulthood. To ensure the peak during the teenage years was not related to more frequent performance of cascade family screening, a proband-only sensitivity analysis was performed, and the pattern remained unchanged.

**Figure 2:**
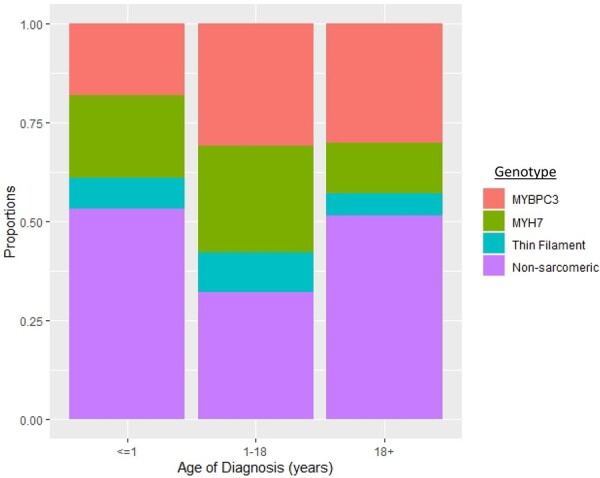
Genotype distribution by age of hypertrophic cardiomyopathy diagnosis. Childhood-onset is predominantly sarcomeric hypertrophic cardiomyopathy, whereas infant- and adult-onset hypertrophic cardiomyopathies are more commonly non-sarcomeric. Of those with sarcomeric hypertrophic cardiomyopathy, *MYH7* variants are more common earlier in life whereas the proportion of patients with *MYBPC3* variants increases with age.

*Figure 1B* highlights the association between age of diagnosis and genotype in the 4746 patients who underwent genetic testing. Although infants are less likely to have sarcomeric disease, patients with sarcomeric HCM are diagnosed an average of 9 years earlier than those with non-sarcomeric HCM (mean of 38 vs. 47 years of age). Sarcomeric HCM is more prevalent than non-sarcomeric until approximately age 50 years, when non-sarcomeric HCM becomes more prevalent. Focusing on sarcomeric HCM, *MYH7* variants are the most common in infant- and childhood-onset HCM. The proportion of *MYBPC3* variants increases with age at diagnosis, such that it is the most common by adulthood (*Figure 2*). A listing of all pathogenic and likely pathogenic variants is presented in Supplementary material online, *Table S1*.

Based on defined age of diagnosis strata, 184 patients (2.4%) were diagnosed in infancy (<1 year old), 1128 (14.7%) in childhood (ages 1–18 years), and 6365 (82.9%) in adulthood (>18 years old). Baseline characteristics for these three ages of diagnosis are displayed in *Table 1*. Patients with infant-onset HCM were the least likely to have a family history of HCM (*P* < 0.001) or have pathogenic/likely pathogenic sarcomere gene variants (*P* < 0.001). Conversely, patients with childhood-onset HCM were the most likely to have a family history (49%, *P* < 0.001) and have sarcomeric disease (64%, *P* < 0.001).

**Table 1:** Baseline characteristics by age of diagnosis

	**Infant-onset HCM <1 year (*n* = 184)**	**Childhood-onset HCM 1**–**18 years (*n* = 1128)**	**Adult-onset HCM >18 years (*n* = 6365)**
Age at diagnosis, years, mean (SD)	0.2 (0.3)	13.1 (4.3)	48.0 (15.3)
Time from diagnosis to baseline SHaRe visit (years)	2.0	2.1	2.6
Male sex, *n* (%)	104 (56.6)	726 (64.4)	3772 (59.3)
Genetic testing performed, *n* (%)	103 (56.0)	686 (60.8)	4049 (63.6)
Sarcomeric, *n* (%)^a^	37 (35.9)	431 (62.8)	1974 (48.8)
Non-sarcomeric, *n* (%)^a^	54 (52.4)	188 (27.4)	1746 (43.1)
Proband, *n* (%)	177 (96.2)	960 (85.1)	5568 (87.5)
Family history of HCM, *n* (%)	48 (26.1)	548 (48.6)	2247 (35.3)
Max LV wall thickness, mm, mean (SD)	8.5 (3.3)	14.6 (7.0)	17.1 (5.6)^b^
Max LV wall thickness, Z-score, mean (SD)	11.8 (9.0)	8.5 (7.2)	7.9 (5.5)
Echo LVEF, % (SD)	73 (15.2)	72 (9.5)	67 (8.0)
LVOT obstruction (gradient >30 mmHg), *n* (%)	78 (42.4)	293 (26.0)	2441 (38.4)
Follow-up time (years), mean (SD)	11.1 (11.8)	12.3 (11.8)	11.4 (10.4)

HCM, hypertrophic cardiomyopathy; LV, left ventricular; LVEF, left ventricular ejection fraction; LVOT, left ventricular outflow tract; SD, standard deviation; SHaRe, Sarcomeric Human Cardiomyopathy Registry.

a % reported is based on the % of patients with genetic testing. Patients with sarcomeric variants of unknown significance are not listed.

b The normal range of LV wall thickness in adults is 6–10 mm.

### Clinical outcomes of infant- vs. childhood-onset hypertrophic cardiomyopathy

Of the 184 patients diagnosed during infancy, morbidity and mortality were initially greater than in those diagnosed during childhood (Supplementary material online, *Figure S1*). However, surviving patients with infant-onset HCM had better long-term outcomes than those diagnosed in childhood. By 5 years after initial SHaRe visit, the childhood-onset group had accrued higher overall event rates than the infant-onset group (Supplementary material online, *Figure S1A*). Ultimately, those diagnosed during childhood experienced more HF and arrhythmias compared with those diagnosed in infancy (Supplementary material online, *Figure S1B–D*).

### Clinical outcomes of childhood-onset hypertrophic cardiomyopathy

**Figure 3:**
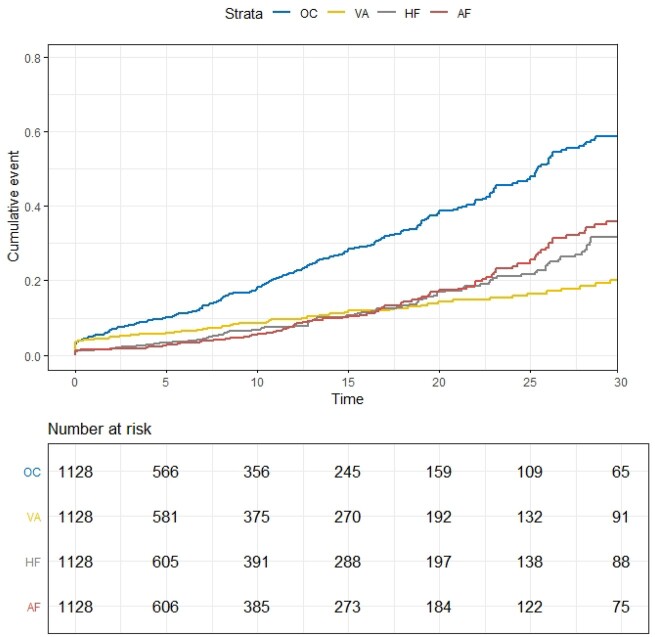
Cumulative incidence of cardiac events in childhood-onset hypertrophic cardiomyopathy. Approximately half of the events in the first 10 years after baseline visit to a Sarcomeric Human Cardiomyopathy Registry site are life-threatening ventricular arrhythmias; atrial fibrillation and heart failure are less common over this timeframe. By 25 years after baseline, nearly 50% of patients reached the overall cardiac composite outcome. Heart failure and atrial fibrillation are more common later in the disease course. AF, atrial fibrillation; HF, heart failure composite outcome; OC, overall composite outcome; VA, ventricular arrhythmia composite outcome.

In the 1128 patients diagnosed with HCM during childhood, the mean age at diagnosis was 13.1 years (*Table 1*). Of these, 64% were male, 49% had a family history, and 85% were probands. In the first decade after initial SHaRe visit, nearly 20% of patients had a cardiac event as reflected by the overall composite endpoint encompassing HF, ventricular arrhythmias, AF, stroke, and all-cause death (*Figure 3* and Supplementary material online, *Table S2*). Nearly half of the events in the first 10 years after baseline visit were life-threatening ventricular arrhythmias [cumulative incidence 8.8% (95% CI 7.1–10.9%)]; AF and HF were less common over this timeframe (Supplementary material online, *Table S2*). Event rates continued to increase over time and by 25 years after HCM baseline visit, nearly 50% of patients had reached the overall cardiac composite outcome [48.9% (95% CI 43.7–54.4%)]. These later cardiac events were more likely to be HF and AF rather than ventricular arrhythmias (*Figure 3* and Supplementary material online, *Table S2*).

Patients with childhood-onset HCM were most likely to have sarcomeric disease. Fifty-three per cent of childhood-onset HCM patients were genotyped and of these, 64% had pathogenic/likely pathogenic sarcomeric variants compared with 49% of genotyped adult-onset and 36% of genotyped infant-onset patients (*Table 1*). The mean age of diagnosis was similar in sarcomeric and non-sarcomeric HCM (12.9 and 12.3, respectively, *P* = 0.85) and both groups were male-predominant (66% and 73%, respectively, *P* = 0.14). Patients with childhood-onset sarcomeric HCM had a 63% increased risk for the overall cardiac composite outcome compared with non-sarcomeric childhood-onset HCM [adjusted HR 1.63 (1.18–2.24), *P* = 0.004] (Supplementary material online, *Figure S2A*). This difference did not emerge until over 10 years after first visit and was driven by HF [HR 2.74 (1.66–4.52), *P* < 0.001] (Supplementary material online, *Figure S2B*) and AF [HR 1.86 (1.12–3.07), *P* = 0.01] (Supplementary material online, *Figure S2D*), with no significant increased hazard identified for the ventricular arrhythmia composite (Supplementary material online, *Figure S2C*), although low event rates limit power.

Potential risk factors for adverse outcomes in childhood-onset HCM were evaluated in a multivariable model that included the presence of a sarcomeric variant, proband status, sex, left ventricular outflow tract obstruction, and age at diagnosis within childhood (*Table 2*). The presence of a sarcomeric variant was associated with a 67% increased risk of overall cardiac composite outcomes [HR_adj_ 1.67 (1.16–2.41), *P* = 0.006] and greater than two-fold increased risk of HF [HR 2.39 (1.36–4.20), *P* = 0.003]. Age at diagnosis (within childhood) was the only significant predictor of AF and had only modest effect. Sex (Supplementary material online, *Figure S3*) and the presence of obstructive physiology were not significant predictors of cardiac events in childhood-onset HCM, including HF or life-threatening ventricular arrhythmias.

**Table 2:** Multivariable model of potential risk predictors in childhood-onset hypertrophic cardiomyopathy

	**Overall composite No. of events: 173 No. of patients: 617**		**VA composite No. of events: 76 No. of patients: 617**		**HF composite No. of events: 90 No. of patients: 617**		**Atrial fibrillation No. of events: 81 No. of patients: 617**	
	HR (95% CI)	*P*-value	HR (95% CI)	*P*-value	HR (95% CI)	*P*-value	HR (95% CI)	*P*-value
Sarcomeric HCM	1.67 (1.16, 2.41)	0.006	1.22 (0.71, 2.08)	0.47	2.39 (1.36, 4.20)	0.003	1.63 (0.93, 2.87)	0.08
Family proband	2.07 (1.21, 3.55)	0.008	1.32 (0.65, 2.70)	0.44	1.82 (0.87, 3.80)	0.11	1.91 (0.87, 4.19)	0.11
Male	0.99 (0.73, 1.34)	0.94	1.03 (0.64, 1.67)	0.89	0.75 (0.49, 1.14)	0.18	0.96 (0.62, 1.50)	0.86
Age at diagnosis	1.01 (0.97, 1.04)	0.73	0.96 (0.91, 1.01)	0.10	1.00 (0.95, 1.04)	0.83	1.08 (1.02, 1.14)	0.009
LVOT obstruction	1.15 (0.82, 1.61)	0.44	0.89 (0.51, 1.52)	0.66	1.41 (0.87, 2.27)	0.16	1.09 (0.66, 1.80)	0.75

Adjusted for sex, sarcomeric variant status, proband status, age at diagnosis, and obstruction. Two patients not included due to missing LVOT data.

CI, confidence interval; HCM, hypertrophic cardiomyopathy; HF, heart failure; HR, hazard ratio; LVOT, left ventricular outflow tract; VA, ventricular arrhythmia.

### Clinical outcomes of childhood-onset vs. adulthood-onset hypertrophic cardiomyopathy

**Figure 4:**
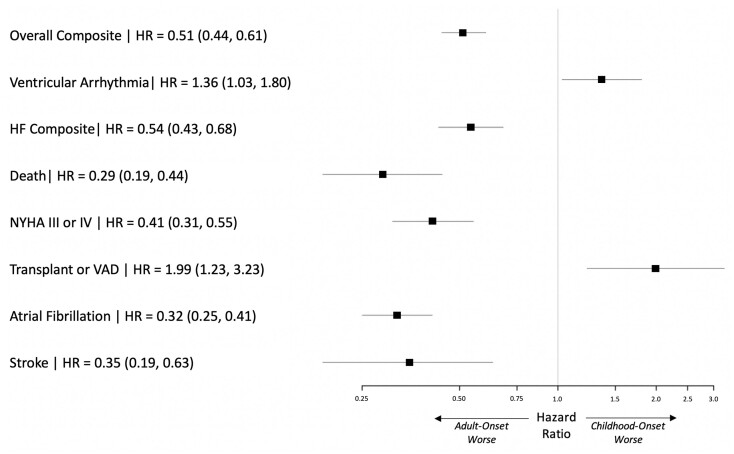
Comparison of outcomes in childhood-onset (*n* = 617) vs. adult-onset hypertrophic cardiomyopathy (*n* = 3779). Childhood-onset hypertrophic cardiomyopathy is associated with 36% increased risk of life-threatening ventricular arrhythmias [HR_adj_ 1.36 (1.03–1.80)] and two-fold risk of requiring transplant or ventricular assist device [HR_adj_ 1.99 (1.23–3.23)] compared with adult-onset hypertrophic cardiomyopathy. Adjusted for sex, sarcomeric variant status, proband status, and age at disgnosis.

Significant differences were identified in comparing outcomes for childhood- and adult-onset HCM **(** *Figure 4* **)**. Analysing patients from the time of first visit, those diagnosed in childhood were 36% more likely to develop life-threatening ventricular arrhythmias [HR_adj_ 1.36 (1.03–1.80)] and twice as likely to require transplant or ventricular assist device [HR_adj_ 1.99 (1.23–3.23)] than patients with adult-onset HCM. Given the broad age range encompassed in adult-onset HCM, we performed sensitivity analyses, dividing the adult population into early (age of diagnosis 19–40 years) and later adult-onset (age of diagnosis >40 years) cohorts. Findings remained consistent when comparing childhood-onset to both early and later adult-onset HCM (Supplementary material online, *Figure S4*). Although sarcomeric HCM was associated with worse outcomes, the impact of sarcomeric variants appeared to differ in childhood- vs. adult-onset HCM. Sarcomeric disease was most strongly associated with the overall composite and HF in childhood-onset HCM (Supplementary material online, *Figure S5*), whereas in adult-onset HCM, sarcomeric disease was most clearly associated with increased risk of all-cause death and AF (Supplementary material online, *Figure S6*).

## Discussion

The goal of this study was to better characterize the natural history of childhood-onset HCM, particularly examining the impact of genetic background (sarcomeric vs. non-sarcomeric disease) and contrasting outcomes to adult-onset disease. We identified four main findings. First, there appear to be three distinct peaks when HCM is diagnosed over the lifespan: infancy, adolescence, and middle adulthood, with childhood-onset HCM being predominantly sarcomeric disease. Second, childhood-onset HCM is clinically different from both infant-onset and adult-onset HCM. Third, patients with childhood-onset HCM are at the highest risk for life-threatening ventricular arrhythmias and requiring advanced HF therapies (*Graphical Abstract*). And finally, the natural history of sarcomeric HCM may differ depending on age of diagnosis with HCM.

Our data, from a diverse, multicentre cohort, indicate that the emergence of phenotypic HCM tends to cluster during infancy, adolescence/early adulthood, and middle adulthood. Moreover, these different ages of diagnosis appear to follow distinct clinical courses. Recent studies have demonstrated that infant-onset HCM carries higher mortality in the 1st year with relatively good outcomes thereafter.^4^ ^,^ ^5^ However, these cohorts included high proportions of patients with RASopathies and inborn errors of metabolism, rather than primary, non-syndromic HCM. Our study excluded these syndromic causes of HCM, thus provided one of the largest non-syndromic infant-onset HCM populations to date. In this more focused cohort, we similarly found that infants with HCM have higher mortality early on, followed by a lower event rate in survivors compared with childhood-onset HCM. Additionally, infants were less likely to have stereotypical sarcomeric HCM. These findings confirm that infant-onset HCM may be a variation of the disease process that should be considered differently in clinical and research settings.

The second two peaks in age of diagnosis occur in the teenage/early adult years and in middle adulthood. These cohorts also appear to have distinct clinical characteristics and outcomes. Specifically, patients diagnosed with HCM in childhood were more likely to have sarcomeric disease, had higher risk for life-threatening ventricular arrhythmias, and were more likely to require advanced therapies for HF. In contrast, patients diagnosed in adulthood, especially those diagnosed after 50 years of age, were more likely to have non-sarcomeric disease and were at greater risk of AF. These differences again indicate that there are clinically relevant subgroups of HCM. Further investigation is required to better characterize disease aetiology and allow more precise diagnoses and patient stratification across the lifespan.

Having set childhood-onset HCM apart from both infant- and adult-onset disease, we sought to gain more specific insights into this cohort. With greater utilization of genetic testing in SHaRe sites (>50% of the overall cohort), we found that the majority (63%) of childhood-onset HCM is due to sarcomeric disease. At 63%, this is a higher prevalence of sarcomeric HCM than previously identified in smaller cohorts or cohorts with lower utilization of genetic testing.^12^ Additionally, males appeared to be disproportionately represented in childhood-onset HCM (64% of the cohort), suggesting earlier penetrance of disease in males, although sex did not significantly affect outcomes. Patients with childhood-onset HCM have a cardiac event rate of ∼2%/year in the first three decades following baseline SHaRe visit and were at particularly increased risk for life-threatening ventricular arrhythmias and need for advanced mechanical support for HF. The risk of ventricular arrhythmia was relatively constant during this period at ∼0.7%/year but represented the most common adverse cardiac event in the first decade after initial visit. The ventricular arrhythmia rate in SHaRe is lower than the 1.5%/year annual rate in another published cohort,^13^ potentially related to differences in patient population, selection biases, and inherent variability due to relatively small sample sizes and rare events. The risk of AF and HF increases with age, such that by the 3rd decade after initial visit, the incidence of AF and HF was greater than ventricular arrhythmias. By the time childhood-onset HCM patients were in their 40s, over 50% experienced a cardiac endpoint, including ∼20% with ventricular arrhythmias, 30% with AF, and nearly 30% with evidence of left ventricular dysfunction or HF.

The association between age of HCM onset, genotype, and outcomes has clinical relevance. Overall, patients with sarcomeric HCM presented an average of 9 years earlier than patients with non-sarcomeric HCM and constituted the majority of patients diagnosed during the teenage years. Sarcomeric HCM appeared to be more common throughout young adulthood but continued to be relatively prevalent until the latter part of the 5th decade, before declining. Conversely, non-sarcomeric HCM was most commonly diagnosed after age 50, with peak age at diagnosis during the 6th decade of life, comprising the bulk of the 3rd peak in age of HCM diagnosis during middle adulthood. Sarcomeric variants have been associated with worse outcomes in HCM.^7^ ^,^ ^14^ ^,^ ^15^ Results from this study extend this finding to childhood-onset HCM, showing a two-fold increased risk of HF for those with sarcomeric disease. The natural history of sarcomeric HCM appeared to differ in childhood-onset vs. adult-onset disease. Children with sarcomeric HCM had a greater risk of HF and the overall composite outcome compared with children with non-sarcomeric HCM, whereas in adults, sarcomeric HCM was associated with a greater risk of AF and all-cause mortality. The underlying mechanisms for these differences are not clear but presumably account for variation in disease expression, comorbidities, and clinical course related to age of onset. Taken together, these results highlight the importance of screening young 1st-degree relatives of patients with HCM^16^ ^,^ ^17^ and for improving identification of children at highest risk for cardiovascular sequelae.^13^ ^,^ ^18^

### Limitations

This study has limitations inherent to observational studies using partially retrospective data, including missing data, and survival bias. Childhood-onset HCM is relatively rare, limiting power to detect differences between subgroups and identify predictors of outcomes. However, our study is the largest study of childhood-onset HCM to date,^13^ even after excluding syndromic causes of HCM and setting aside infant-onset disease. Our study draws from adult HCM centres and therefore may be more representative of childhood-onset HCM patients who survived to adulthood, however, ∼40% of our childhood-onset patients originated from paediatric cardiology SHaRe sites. There may also be survival bias as patients who died or had major adverse events prior to referral to a SHaRe site would not be captured. However, the median time from HCM diagnosis to initial SHaRe visit in the childhood-onset group was only 2.1 years. Furthermore, patients diagnosed later in adulthood have more comorbidities and risk of AF, HF, and death from non-HCM related causes, leading to more competing risks than are present in childhood-onset HCM.

## Conclusions

Patients with childhood-onset HCM have important differences in clinical characteristics, genotype, and cardiovascular outcomes. They are more likely to have sarcomeric disease, have a higher risk of ventricular arrhythmias, and a greater frequency of advanced HF than patients diagnosed during adulthood. These findings help improve patient stratification and underscore the need to better understand disease biology.

## Supplementary material

Supplementary material is available at *European Heart Journal* online.

## Data availability

The SHaRe Registry data is not universally available but we encourage parties interested in collaborating to contact the corresponding author directly.

##  

**Conflict of interest:** N.A.M. reports involvement in clinical trials with Amgen, Pfizer, Novartis, and AstraZeneca without personal fees, payments, or increase in salary. L.H., E.A.A., A.C.P., C.S., S.D.C., and S.G.W. have nothing to disclose. I.O. reports grants and personal fees from MyoKardia, Genzyme, Shire, and Amicus; personal fees from Cytokinetics; grants from Bayer and Menarini International, outside the submitted work. S.M.D. reports grants from MyoKardia, during the conduct of the study. M.M. reports personal fees from MyoKardia, outside the submitted work. J.I. reports grants from MyoKardia, Inc. during the conduct of the study. D.J. reports grants from MyoKardia, during the conduct of the study; grants and personal fees from MyoKardia, personal fees from Cytokinetics, outside the submitted work. J.W.R. reports personal fees from Amgen, Abiomed, Novartis, Cytokinetics, MyoKardia, and Bayer, outside the submitted work. J.S.W. reports grants and non-financial support from the Wellcome Trust [107469/Z/15/Z], Medical Research Council (UK), British Heart Foundation, NIHR Royal Brompton Cardiovascular Biomedical Research Unit, and the NIHR Imperial College Biomedical Research Centre, during the conduct of the study; grants and personal fees from MyoKardia, outside the submitted work. S.S. reports personal fees from MyoKardia, Inc., from null, outside the submitted work. A.S.H. reports grants from MyoKardia, during the conduct of the study; personal fees from Tenaya Therapeutics, outside the submitted work. C.Y.H. reports grants and personal fees from MyoKardia, personal fees from Novartis and Tenaya, during the conduct of the study.

## Supplementary Material

ehab148_Supplementary_Appendix
