# Supplementary material for: Clinical characteristics and outcomes in childhood-onset hypertrophic cardiomyopathy
Source: Eur Heart J. 2021 Mar 26;42(20):1988–96. doi: 10.1093/eurheartj/ehab148 (PMC8139852; doi:10.1093/eurheartj/ehab148)

Supplemental Appendix

Supplemental Table 1: List of Pathogenic and Likely Pathogenic Variants from Each Gene

Gene	c.designation	p.designation	Classification	Count
ACTC1	c.275_277del	p.Phe92del	LP	1
ACTC1	c.281A>G	p.Asn94Ser	LP	1
ACTC1	c.301G>A	p.Glu101Lys	LP	1
ACTC1	c.694G>A		P	1
ACTC1	c.695C>T	p.Ala232Val	P	2
ACTC1	c.69T>A	p.Phe23Leu	P	1
ACTC1	c.968C>T	p.Ala323Val	LP	2
ACTC1	c.992T>A	p.Ile331Asn	P	1
MYBPC3	c.1000G>T	p.Glu334*	P	3
MYBPC3	c.1012G>A	p.Val388Met	LP	1
MYBPC3	c.1015C>T	p.Gln339*	P	1
MYBPC3	c.1020C>G	p.Tyr340*	P	6
MYBPC3	c.1053_1054delinsTT		P	2
MYBPC3	c.1090+453C>T		LP	3
MYBPC3	c.1090G>A	p.Ala364Thr	P	6
MYBPC3	c.1091-?_2308+?Del	p.Phe365-Asp770del	P	2
MYBPC3	c.1112C>G	p.Pro371Arg	LP	11
MYBPC3	c.1153_1168del	p.Val385Metfs*16	P	3
MYBPC3	c.1168del	p.His390Metfs*16	P	1
MYBPC3	c.1171del	p.Asp391Thrfs*15	P	1
MYBPC3	c.1174del	p.Ala392Leufs*14	P	14
MYBPC3	c.1210C>T	p.Gln404*	P	7
MYBPC3	c.1224-2A>G		P	2
MYBPC3	c.1227-13G>A		P	2
MYBPC3	c.1227-1G>C		P	1
MYBPC3	c.1227-2A>G		P	1
MYBPC3	c.1235_1236del	p.Phe412*	P	4
MYBPC3	c.1351+2T>C		P	1
MYBPC3	c.1354_1355insC	p.Val454Cysfs*21	P	1
MYBPC3	c.1359del	p.Val454Cysfs*12	P	2
MYBPC3	c.1377del	p.Leu460Trpfs*6	P	1
MYBPC3	c.1404del	p.Gln469fs	P	1
MYBPC3	c.1456T>G	p.Trp486Gly	LP	1
MYBPC3	c.1458-1G>A		P	11
MYBPC3	c.1458-1G>C		P	10
MYBPC3	c.1458-6G>A		LP	1
MYBPC3	c.1483C>G	p.Arg495Gly	P	5
MYBPC3	c.1483C>T	p.Arg495Trp	P	3
MYBPC3	c.1484G>A	p.Arg495Gln	P	25
MYBPC3	c.1504C>T	p.Arg502Trp	LP	55
MYBPC3	c.1505G>A	p.Arg502Gln	P	34
MYBPC3	c.1509C>G	p.Phe503Leu	P	2
MYBPC3	c.1575T>G	p.Tyr525*	P	2
MYBPC3	c.1591G>C	p.Gly531Arg	LP	9
MYBPC3	c.1624+1G>A		P	6
MYBPC3	c.1624+4A>T		P	14
MYBPC3	c.1624G>C	p.Glu542Gln	P	28
MYBPC3	c.162del	p.Lys54Asnfs*13	P	1
MYBPC3	c.1678del	p.Asp560Thrfs*19	P	1
MYBPC3	c.1687del	p.Val563Cysfs*16	P	1
MYBPC3	c.1729del	p.Trp577Alafs*27	P	1
MYBPC3	c.1755del	p.Asp587Thrfs*4	P	1

MYBPC3	c.177_187del	p.Glu60fs*49	P	4
MYBPC3	c.1789C>T	p.Arg597Trp	LP	4
MYBPC3	c.1790G>A	p.Arg597Gln	LP	2
MYBPC3	c.1791-1G>A		P	1
MYBPC3	c.1800del	p.Lys600Asnfs*2	P	3
MYBPC3	c.1838dup	p.Asp613Glufs*25	P	2
MYBPC3	c.1892del	p.Phe631fs	P	3
MYBPC3	c.1895del	p.Met632Argfs	P	7
MYBPC3	c.1897+5G>A		LP	2
MYBPC3	c.1898-1G>A		P	1
MYBPC3	c.1900del	p.Val634Serfs*29	P	1
MYBPC3	c.1928-2A>G		P	24
MYBPC3	c.1999_2000delinsG	p.Leu667Thrfs*25	P	2
MYBPC3	c.2025_2026del	p.Asp676Profs*16	P	1
MYBPC3	c.2048G>A	p.Trp683*	P	1
MYBPC3	c.2073del	p.Asn691fs	P	1
MYBPC3	c.208del	p.Glu70fs*25	P	1
MYBPC3	c.2096del	p.Pro699Glnfs*55	P	4
MYBPC3	c.2113dup	p.Thr705Asnfs*3	P	2
MYBPC3	c.2132G>A	p.Trp711*	P	1
MYBPC3	c.2148+158_2737+258del3548insT		P	1
MYBPC3	c.2149-1G>A		P	2
MYBPC3	c.2149-2del		P	2
MYBPC3	c.2149-9C>A		P	5
MYBPC3	c.2156A>G	p.Arg719Gln	P	1
MYBPC3	c.2157_2158del	p.Cys719*	P	1
MYBPC3	c.2182G>T	p.Glu728*	P	1
MYBPC3	c.2221del	p.Ala741Glnfs*13	P	1
MYBPC3	c.2240_2245delinsCCTTC	p.Gly747Alafs*7	P	1
MYBPC3	c.2258dup	p.Lys754Glufs*79	P	10
MYBPC3	c.2259_260insTC	p.Lys754Serfs*69	LP	1
MYBPC3	c.2267del	p.Pro756Leufs*66	LP	1
MYBPC3	c.2267del	p.Pro756Leufs*66	P	1
MYBPC3	c.227_228insA	p.Ser78Ilefs*35	P	1
MYBPC3	c.2274C>T	p.Gly758=	LP	3
MYBPC3	c.2302G>A		P	1
MYBPC3	c.2304_2308+2del	p.Ile769Argfs*62	P	1
MYBPC3	c.2308+1G>A		P	4
MYBPC3	c.2308+1G>T		P	4
MYBPC3	c.2308+2T>G		P	1
MYBPC3	c.2308G>A		P	9
MYBPC3	c.2309-2A>G		P	13
MYBPC3	c.2371C>T	p.Gln791*	P	1
MYBPC3	c.2373insG	p.Trp792Valfs*41	P	142
MYBPC3	c.2374insG	p.Trp792fs	P	1
MYBPC3	c.2374T>C	p.Trp792Arg	LP	15
MYBPC3	c.2376G>A	p.Trp792Valfs*41	P	1
MYBPC3	c.237C>G	p.Tyr79*	P	1
MYBPC3	c.2391C>A	p.Tyr797*	P	6
MYBPC3	c.2413+1G>A		P	1
MYBPC3	c.2414-2A>G		P	1
MYBPC3	c.2429G>A	p.Arg810His	LP	15
MYBPC3	c.2429G>T	p.Arg810Leu	LP	4
MYBPC3	c.2454G>A	p.Trp818*	P	2
MYBPC3	c.2459G>A	p.Arg820Gln	LP	2
MYBPC3	c.2490dup	p.His831Serfs*2	P	1

MYBPC3	c.25+1G>A		P	2
MYBPC3	c.2524_2525insT		P	1
MYBPC3	c.2524:G>A	p.Trp818*	P	1
MYBPC3	c.2524G>A		P	1
MYBPC3	c.2526C>G	p.Tyr842*	P	1
MYBPC3	c.2541C>G	p.Tyr847*	P	2
MYBPC3	c.2543_2544dup		P	1
MYBPC3	c.2550del	p.Asn850Lysfs*29	P	2
MYBPC3	c.2554insT	p.Ile852fs*	P	2
MYBPC3	c.2555_2556insT	p.Gly853fs	P	1
MYBPC3	c.2558del		P	2
MYBPC3	c.2573G>A	p.Ser858Asn	LP	4
MYBPC3	c.26-2A>G		P	3
MYBPC3	c.2603-1G>A		P	1
MYBPC3	c.2604-2605delTCinsA	p.Ser871Alafs*8	P	1
MYBPC3	c.2670_2671insG	p.Arg891Alafs*160	P	3
MYBPC3	c.2670G>A	p.Trp890*	P	9
MYBPC3	c.2689_2698del	p.Gly897Alafs*24	P	6
MYBPC3	c.2710del	p.Tyr904Thrfs*20	P	6
MYBPC3	c.2735del	p.Gly912Alafs*12	P	4
MYBPC3	c.2737+2T>A		P	1
MYBPC3	c.2737+5G>A		P	1
MYBPC3	c.2738-2A>T		LP	1
MYBPC3	c.2747G>A	p.Trp916*	P	3
MYBPC3	c.2780_2781del	p.Thr927Ilefs*123	P	1
MYBPC3	c.2792dup	p.Lys932Glufs*119	P	1
MYBPC3	c.2827C>T	p.Arg943*	P	68
MYBPC3	c.2833_2834del	p.Arg945Glyfs*105	P	1
MYBPC3	c.2846dup	p.Met949Ilefs*102	P	1
MYBPC3	c.2862_2865del		P	2
MYBPC3	c.2864_2865del	p.Pro955Argfs*95	P	54
MYBPC3	c.2864del	p.Pro955Argfs*95	LP	7
MYBPC3	c.2869dup	p.Thr957fs*94	P	1
MYBPC3	c.2893C>T	p.Gln965*	P	1
MYBPC3	c.2905+1G>A		P	9
MYBPC3	c.2905+5G>T		P	1
MYBPC3	c.2905C>T	p.Gln969*	P	8
MYBPC3	c.292G>C	p.Glu98*	P	1
MYBPC3	c.2943_2947del	p.Gln981fs	P	2
MYBPC3	c.2992C>T	p.Gln998*	P	2
MYBPC3	c.2994+2T>C		P	3
MYBPC3	c.3029del	p.Glu1010fs	P	1
MYBPC3	c.3040del	p.Leu1014Trpfs*6	P	1
MYBPC3	c.306_306del	p.Met103Cysfs*56	P	1
MYBPC3	c.3065G>C	p.Arg1022Pro	LP	4
MYBPC3	c.3100del	p.Ala1034Profs*12	P	1
MYBPC3	c.3124_3125insAA	p.Thr1042Lysfs*5	P	2
MYBPC3	c.3127dup	p.Tyr1043Leufs*8	P	1
MYBPC3	c.3129C>A	p.Tyr1043*	P	1
MYBPC3	c.3166dup	p.Lys1055*	LP	1
MYBPC3	c.3181C>T	p.Gln1061*	P	2
MYBPC3	c.3190+1G>A		P	3
MYBPC3	c.3190+2T>G		LP	1
MYBPC3	c.3190+5G>A		P	7
MYBPC3	c.3192dup	p.Lys1065Glufs*12	P	38
MYBPC3	c.3217dup	p.Arg1073Profs*4	P	1

MYBPC3	c.3226_3227insT	p.Asp1076Valfs*6	P	2
MYBPC3	c.3233G>A	p.Trp1078*	P	6
MYBPC3	c.3286G>T	p.Glu1096*	P	3
MYBPC3	c.3288del		P	1
MYBPC3	c.3288G>A	p.Glu1096*	P	3
MYBPC3	c.3293G>A	p.Trp1098*	P	1
MYBPC3	c.3297dup	p.Tyr1100Valfs*49	P	1
MYBPC3	c.3300C>A	p.Tyr1100*	P	1
MYBPC3	c.3321dup	p.Lys1108Glufs*41	P	1
MYBPC3	c.3330+2T>C		P	2
MYBPC3	c.3330+2T>G		P	16
MYBPC3	c.3330+5G>A		P	1
MYBPC3	c.3330+5G>C		P	7
MYBPC3	c.3330+T>G		P	1
MYBPC3	c.3331-1G>A		P	2
MYBPC3	c.3331-1G>C		P	3
MYBPC3	c.3331-2A>G		P	1
MYBPC3	c.3332_3335dup	p.Trp1112Leufs*37	P	1
MYBPC3	c.3334dup	p.Trp1112Leufs*37	P	1
MYBPC3	c.3335G>A	p.Trp1112*	P	1
MYBPC3	c.3350+5G>C		P	1
MYBPC3	c.3372C>A	p.Cys1124*	P	2
MYBPC3	c.3414_3415insC	p.Val1139Argfs*10	P	2
MYBPC3	c.3432_3435dup	p.Phe1147Trpfs*3	P	2
MYBPC3	c.3467dup	p.Pro1157Alafs*12	P	1
MYBPC3	c.3476_3477insATTT	p.Phe1159Leufs*11	P	1
MYBPC3	c.3490+1G>A		P	2
MYBPC3	c.3490+1G>T		P	3
MYBPC3	c.3490+5G>A		P	1
MYBPC3	c.3514_3517dup	p.Lys1173Ilefs*2	P	2
MYBPC3	c.3617_3618del	p.Gly1206Glufs*35	P	1
MYBPC3	c.3617del	p.Gly1206Valfs*31	P	5
MYBPC3	c.3624_3625insC	p.Lys1209Glnfs*33	P	5
MYBPC3	c.3624del	p.Lys1209Glnfs*28	P	8
MYBPC3	c.3627+1G>A		P	1
MYBPC3	c.3627+2T>G		P	1
MYBPC3	c.3628-1G>A		P	1
MYBPC3	c.3628-41_3628-17del		P	10
MYBPC3	c.362del	p.Pro121Argfs*38	P	1
MYBPC3	c.3642G>A	p.Trp1214*	P	1
MYBPC3	c.3662_3662del	p.Leu1221Argfs*16	P	2
MYBPC3	c.3690_3691del	p.Phe1230Leufs*11	P	1
MYBPC3	c.3694A>T	p.Lys1232*	P	1
MYBPC3	c.3697C>T	p.Gln1233*	P	21
MYBPC3	c.3697C>T	p.Gln1233*	P	1
MYBPC3	c.3702_3703del	p.Leu1236fs	P	1
MYBPC3	c.3712_3713del	p.Leu1238Glyfs*3	P	1
MYBPC3	c.3713T>C	p.Leu1238Pro	LP	1
MYBPC3	c.3742_3759dup	p.Gly1248_Cys1253dup	P	7
MYBPC3	c.3742_3759dup18	p.Gly1248_cys1253Dup	P	1
MYBPC3	c.3767_3768del	p.Thr1256Lysfs*9	P	2
MYBPC3	c.3771C>A	p.Asn1257Lys	LP	2
MYBPC3	c.3776del	p.Gln1259fs	P	10
MYBPC3	c.3811C>T	p.Arg1271*	P	3
MYBPC3	c.3814+1G>A		P	1
MYBPC3	c.3815-1G>A		P	1

MYBPC3	c.3G>C	p.Met1?	P	1
MYBPC3	c.3G>Exon1	p.Met1?	P	1
MYBPC3	c.407-1G>A		P	1
MYBPC3	c.410C>G	p.Ser137*	P	1
MYBPC3	c.436_437insA	p.Thr146fs	P	1
MYBPC3	c.459del	p.Ile154Leufs*5	P	4
MYBPC3	c.481C>T	p.Pro161Ser	P	9
MYBPC3	c.505+1G>A		P	1
MYBPC3	c.506-1G>A		P	1
MYBPC3	c.506-2A>C		P	4
MYBPC3	c.540_559del	p.Ala181Cysfs*53	P	1
MYBPC3	c.551dup	p.Lys185Glufs*56	P	2
MYBPC3	c.553A>T	p.Lys185*	P	1
MYBPC3	c.613C>T	p.Gln205*	P	1
MYBPC3	c.65_66insG	p.Ala23Argfs*26	P	1
MYBPC3	c.654+1G>A		P	4
MYBPC3	c.655-?_*26=?Del	p.Val219-Gln1274del	P	1
MYBPC3	c.655-1G>A		P	1
MYBPC3	c.655G>C	p.Val219Leu	P	6
MYBPC3	c.676_701dup26	p.Gly235fs	P	1
MYBPC3	c.688del	p.Gln230fs	P	3
MYBPC3	c.710A>C	p.Tyr237Ser	P	1
MYBPC3	c.772G>A	p.Glu258Lys	P	155
MYBPC3	c.772G>Ap.Glu258Lys,Exon6		P	1
MYBPC3	c.772G>C		P	1
MYBPC3	c.821+1G>A		P	12
MYBPC3	c.821+1G>C		P	1
MYBPC3	c.840_851+5del17		P	1
MYBPC3	c.897del	p.Lys301fs	P	2
MYBPC3	c.913_914del	p.Phe305Profs*27	P	17
MYBPC3	c.927-10C>A		LP	1
MYBPC3	c.927-10C>T		LP	2
MYBPC3	c.927-1G>C		P	1
MYBPC3	c.927-2A>G		P	8
MYBPC3	c.927-9G>A		P	24
MYBPC3	c.932C>A	p.Ser311*	P	1
MYBPC3	c.95del	p.Glu32Glyfs*7	P	1
MYBPC3	c.966G>A	p.Trp322*	P	1
MYBPC3	c.989del	p.Pro330Hisfs*20	P	1
MYBPC3	c.993dup	p.Glu332*	P	1
MYBPC3		p.Leu1221Argfs*16	P	1
MYH7	c.1012G>A	p.Val338Met	LP	4
MYH7	c.1013T>C	p.Val338Ala	LP	1
MYH7	c.1063G>A	p.Ala355Thr	P	2
MYH7	c.1193G>A	p.Gly398Glu	LP	2
MYH7	c.1193G>A	p.Gly398Glu	P	1
MYH7	c.1207C>T	p.Arg403Trp	P	5
MYH7	c.1208G>A	p.Arg403Gln	LP	1
MYH7	c.1208G>A	p.Arg403Gln	P	20
MYH7	c.1324C>T	p.Arg442Cys	P	8
MYH7	c.1331A>G	p.Asn444Ser	P	1
MYH7	c.1357C>T	p.Arg453Cys	LP	3
MYH7	c.1357C>T	p.Arg453Cys	P	11
MYH7	c.1358G>A	p.Arg453His	LP	1
MYH7	c.1358G>A	p.Arg453His	P	1
MYH7	c.1370T>C	p.Ile457Thr	LP	2

MYH7	c.1436A>G	p.Asn479Ser	P	1
MYH7	c.1477A>G	p.Met493Val	P	1
MYH7	c.1479G>C	p.Met493Ile	LP	1
MYH7	c.1491G>T	p.Glu497Asp	LP	3
MYH7	c.1588A>G	p.Ile530Val	LP	1
MYH7	c.1615A>C	p.Met539Leu	P	2
MYH7	c.161G>A	p.Arg54Gln	P	1
MYH7	c.1711G>C	p.Gly571Arg	P	2
MYH7	c.1727A>G	p.His576Arg	LP	4
MYH7	c.1727A>G	p.His576Arg	P	2
MYH7	c.1742A>G	p.His581Arg	LP	3
MYH7	c.1750G>A	p.Gly584Ser	P	4
MYH7	c.1750G>C	p.Gly584Arg	LP	5
MYH7	c.1750G>C	p.Gly584Arg	P	14
MYH7	c.1759G>C	p.Asp587His	LP	1
MYH7	c.1801C>T	p.Leu601Phe	LP	1
MYH7	c.1807G>A	p.Glu603Lys	LP	1
MYH7	c.1816G>A	p.Val606Met	P	15
MYH7	c.1820G>A	p.Gly607Asp	LP	3
MYH7	c.1826A>G	p.Tyr609Cys	LP	1
MYH7	c.1954A>G	p.Arg652Gly	LP	6
MYH7	c.1954A>G	p.Arg652Gly	P	5
MYH7	c.1987C>T	p.Arg663Cys	LP	7
MYH7	c.1987C>T	p.Arg663Cys	P	16
MYH7	c.1988G>A	p.Arg663His	LP	3
MYH7	c.1988G>A	p.Arg663His	P	30
MYH7	c.2011C>T	p.Arg671Cys	P	2
MYH7	c.2069T>C	p.Met690Thr	LP	2
MYH7	c.2069T>G	p.Met690Thr	P	1
MYH7	c.2080C>T	p.Arg694Cys	LP	2
MYH7	c.2080C>T	p.Arg694Cys	P	7
MYH7	c.2087A>G	p.Asn696Ser	LP	1
MYH7	c.208A>T	p.Thr70Ser	LP	1
MYH7	c.2093T>C	p.Val698Ala	P	2
MYH7	c.2104A>G	p.Ile702Val	LP	1
MYH7	c.2104A>G	p.Ile702Val	P	1
MYH7	c.2123G>C	p.Gly708Ala	LP	2
MYH7	c.2129C>A	p.Pro710His	P	3
MYH7	c.2146G>A	p.Gly716Arg	LP	3
MYH7	c.2146G>A	p.Gly716Arg	P	7
MYH7	c.2155C>G	p.Arg719Gly	P	1
MYH7	c.2155C>T	p.Arg719Trp	P	8
MYH7	c.2156G>A	p.Arg719Gln	P	15
MYH7	c.2156G>C	p.Arg719Pro	LP	1
MYH7	c.2167C>G	p.Arg723Gly	LP	4
MYH7	c.2167C>G	p.Arg723Gly	P	1
MYH7	c.2167C>T	p.Arg723Cys	LP	1
MYH7	c.2167C>T	p.Arg723Cys	P	16
MYH7	c.2191C>G	p.Pro731Ala	LP	1
MYH7	c.2191C>T	p.Pro731Ser	LP	4
MYH7	c.2198G>A	p.Gly733Glu	P	2
MYH7	c.2201A>C	p.Gln734Pro	LP	1
MYH7	c.2207T>C	p.Ile736Thr	LP	4
MYH7	c.2207T>C	p.Ile736Thr	P	4
MYH7	c.2221G>A	p.Gly741Arg	LP	1
MYH7	c.2221G>A	p.Gly741Arg	P	5

MYH7	c.2221G>C	p.Gly741Arg	LP	3
MYH7	c.2221G>C	p.Gly741Arg	P	2
MYH7	c.2221G>T	p.Gly741Trp	LP	2
MYH7	c.2221G>T	p.Gly741Trp	P	7
MYH7	c.2291T>A	p.Phe764Tyr	LP	1
MYH7	c.2302G>A	p.Gly768Arg	LP	1
MYH7	c.2302G>A	p.Gly768Arg	P	20
MYH7	c.2306C>T		P	1
MYH7	c.2346C>A	p.Ser782Arg	LP	5
MYH7	c.2347C>T	p.Arg783Cys	LP	1
MYH7	c.2348G>A	p.Arg783His	LP	1
MYH7	c.2389G>A	p.Ala797Thr	P	24
MYH7	c.2420G>A	p.Arg807His	LP	1
MYH7	c.2432T>C	p.Leu811Pro	LP	1
MYH7	c.2471T>C	p.Val824Ala	LP	1
MYH7	c.2524A>G	p.Ser842Gly	LP	1
MYH7	c.2539_2541del	p.Lys847del	LP	5
MYH7	c.2539A>G	p.Lys847Glu	LP	2
MYH7	c.2539A>G	p.Lys847Glu	P	1
MYH7	c.2542_2544del	p.Glu848del	LP	1
MYH7	c.2543A>G	p.Glu848Gly	LP	1
MYH7	c.2558del	p.Gly853fs	P	1
MYH7	c.2572C>A	p.Arg858Ser	P	1
MYH7	c.2572C>T	p.Arg858Cys	LP	1
MYH7	c.2573G>C	p.Arg858Pro	LP	1
MYH7	c.2593A>G	p.Lys865Glu	LP	2
MYH7	c.2594A>G	p.Lys865Arg	LP	3
MYH7	c.2605C>T	p.Arg869Cys	LP	1
MYH7	c.2606G>A	p.Arg869His	LP	5
MYH7	c.2606G>A	p.Arg869His	P	46
MYH7	c.2608C>T	p.Arg870Cys	LP	1
MYH7	c.2609G>A	p.Arg870His	P	4
MYH7	c.2609G>T	p.Arg870Leu	LP	1
MYH7	c.2623_2625del	p.Glu875del	LP	1
MYH7	c.2627_2629del	p.Lys876del	LP	1
MYH7	c.2631G>T	p.Met877Ile	P	1
MYH7	c.2644C>G	p.Gln882Glu	LP	4
MYH7	c.2655T>A	p.Asn885Lys	LP	1
MYH7	c.2681A>G	p.Glu894Gly	LP	12
MYH7	c.2717A>G	p.Asp906Gly	P	6
MYH7	c.2722C>G	p.Leu908Val	P	12
MYH7	c.2723T>A	p.Leu908Gln	P	2
MYH7	c.2761G>A	p.Glu921Lys	LP	1
MYH7	c.2770G>A	p.Glu924Lys	LP	1
MYH7	c.2770G>A	p.Glu924Lys	P	3
MYH7	c.2782G>A	p.Asp928Asn	P	1
MYH7	c.2783A>T	p.Asp928Val	P	2
MYH7	c.2788G>C	p.Glu930Gln	P	2
MYH7	c.2893G>A	p.Glu965Lys	LP	2
MYH7	c.2974C>A	p.Leu992Met	LP	1
MYH7	c.3100-2A>C		P	1
MYH7	c.3133C>T	p.Arg1045Cys	LP	2
MYH7	c.3133C>T	p.Arg1045Cys	P	2
MYH7	c.3134G>A	p.Arg1045His	P	1
MYH7	c.3170G>A	p.Gly1057Asp	P	2
MYH7	c.3325A>G	p.Lys1109Glu	LP	2

MYH7	c.3346G>A	p.Glu1116Lys	P	2
MYH7	c.3358G>A	p.Glu1120Lys	LP	1
MYH7	c.341T>C	p.Ile114Thr	LP	1
MYH7	c.3455A>T	p.Glu1152Val	LP	1
MYH7	c.350A>T	p.Tyr117Phe	LP	1
MYH7	c.3645G>C	p.Gln1215His	LP	1
MYH7	c.4031G>A	p.Arg1344Gln	LP	2
MYH7	c.4052C>T	p.Thr1351Met	P	1
MYH7	c.4066G>A	p.Glu1356Lys	LP	6
MYH7	c.4124A>G	p.Tyr1375Cys	LP	1
MYH7	c.4130C>T	p.Thr1377Met	LP	16
MYH7	c.4135G>A	p.Ala1379Thr	P	6
MYH7	c.428G>A	p.Arg143Gln	LP	4
MYH7	c.428G>A	p.Arg143Gln	P	4
MYH7	c.4363G>T	p.Glu1455*	P	1
MYH7	c.438G>T	p.Lys146Asn	P	2
MYH7	c.4402G>A	p.Glu1468Lys	LP	1
MYH7	c.4487A>C	p.Glu1496Ala	P	1
MYH7	c.4571A>G	p.His1524Arg	LP	1
MYH7	c.4807G>A		P	1
MYH7	c.4992C>A	p.Asn1664Lys	LP	1
MYH7	c.505A>G	p.Arg169Gly	LP	2
MYH7	c.5134C>T	p.Arg1712Trp	LP	2
MYH7	c.5135G>A	p.Arg1712Gln	LP	13
MYH7	c.5305C>A	p.Leu1769Met	P	1
MYH7	c.530C>T	p.Thr177Ile	P	1
MYH7	c.5344A>G	p.Met1782Val	LP	1
MYH7	c.5606A>G	p.Asp1869Gly	LP	1
MYH7	c.5696T>C	p.Val1899Ala	LP	1
MYH7	c.596C>T	p.Ala199Val	P	2
MYH7	c.611G>A	p.Arg204His	LP	8
MYH7	c.676G>A	p.Ala226Thr	LP	1
MYH7	c.677C>T	p.Ala226Val	P	8
MYH7	c.697G>T	p.Ala233Ser	LP	2
MYH7	c.715G>A	p.Asp239Asn	LP	2
MYH7	c.715G>A	p.Asp239Asn	P	1
MYH7	c.745C>G	p.Arg249Gly	P	1
MYH7	c.746G>A	p.Arg249Gln	LP	2
MYH7	c.746G>A	p.Arg249Gln	P	7
MYH7	c.755T>G	p.Phe252Cys	LP	1
MYH7	c.788T>C	p.Ile263Thr	LP	7
MYH7	c.809A>G	p.Lys270Arg	LP	3
MYH7	c.872C>T	p.Ser291Phe	LP	1
MYH7	c.909C>G	p.Ile303Met	LP	1
MYH7	c.920C>A	p.Pro307His	LP	1
MYH7	c.950A>G	p.Glu317Gly	LP	1
MYH7	c.958G>A	p.Val320Met	LP	10
MYH7	c.968T>A	p.Ile323Asn	LP	3
MYH7		p.Arg452His	P	1
MYL2	c.125G>A	p.Gly42Asp	LP	2
MYL2	c.173G>A	p.Arg58Gln	LP	1
MYL2	c.173G>A	p.Arg58Gln	P	8
MYL2	c.184A>T	p.Lys62*	LP	1
MYL2	c.184A>T	p.Lys62*	P	1
MYL2	c.193G>A	p.Glu65Lys	LP	1
MYL2	c.239C>A	p.Thr80Asn	LP	3

MYL2	c.286G>A	p.Glu96Lys	LP	1
MYL2	c.45_46delinsT	p.Asn16Thrfs*34	LP	1
MYL2	c.482A>G	p.His161Arg	LP	1
MYL2	c.484G>A	p.Gly162Arg	P	1
MYL2	c.58A>C	p.Met20Leu	LP	3
MYL2	c.58A>C	p.Met20Leu	P	1
MYL2	c.64G>A	p.Glu22Lys	LP	2
MYL2	c.64G>A	p.Glu22Lys	P	8
MYL3	c.170C>G	p.Ala57Gly	P	1
MYL3	c.435dup	p.Gly146Trpfs*7	LP	1
MYL3	c.445A>G	p.Met149Val	P	1
MYL3	c.452C>T	p.Ala151Val	P	3
MYL3	c.461:G>A	p.Arg154His	P	1
MYL3	c.463C>G	p.His155Asp	LP	1
TNNI3	c.114dup	p.Ser39fs	P	1
TNNI3	c.257_258del	p.Ala86Glyfs*23	P	2
TNNI3	c.356C>A	p.Thr119Asn	LP	1
TNNI3	c.407G>A	p.Arg136Gln	P	1
TNNI3	c.422G>A	p.Arg141Gln	LP	1
TNNI3	c.422G>A	p.Arg141Gln	P	2
TNNI3	c.433C>G	p.Arg145Gly	P	2
TNNI3	c.433C>T	p.Arg145Trp	LP	2
TNNI3	c.433C>T	p.Arg145Trp	P	10
TNNI3	c.434G>A	p.Arg145Gln	LP	2
TNNI3	c.434G>A	p.Arg145Gln	P	1
TNNI3	c.470C>T	p.Ala157Val	P	5
TNNI3	c.484C>T	p.Arg162Trp	LP	5
TNNI3	c.485G>A	p.Arg162Gln	LP	3
TNNI3	c.485G>A	p.Arg162Gln	P	6
TNNI3	c.485G>C	p.Arg162Pro	LP	1
TNNI3	c.485G>C	p.Arg162Pro	P	1
TNNI3	c.497C>T	p.Ser166Phe	LP	2
TNNI3	c.497C>T	p.Ser166Phe	P	2
TNNI3	c.509G>A	p.Arg170Gln	LP	1
TNNI3	c.509G>A	p.Arg170Gln	P	1
TNNI3	c.526G>A	p.Val176Met	LP	1
TNNI3	c.549G>T	p.Lys183Asn	LP	1
TNNI3	c.557G>A	p.Arg186Gln	LP	3
TNNI3	c.557G>A	p.Arg186Gln	P	13
TNNI3	c.568G>T	p.Asp190Tyr	LP	1
TNNI3	c.575G>A	p.Arg192His	P	2
TNNI3	c.575G>T	p.Arg192Leu	LP	1
TNNI3	c.581A>G	p.Asn194Ser	LP	1
TNNI3	c.586G>A	p.Asp196Asn	LP	1
TNNI3	c.592C>G	p.Leu198Val	LP	13
TNNI3	c.592C>T	p.Leu198Val	LP	1
TNNI3	c.611G>A	p.Arg204His	P	2
TNNI3	c.61C>T	p.Arg21Cys	P	1
TNNI3	c.626A>C	p.Glu209Ala	P	1
TNNI3		p.Leu197Val	P	1
TNNT2	c.196A>C	p.Lys66Gln	LP	1
TNNT2	c.236T>A	p.Ile79Asn	P	16
TNNT2	c.274C>T	p.Arg92Trp	P	9
TNNT2	c.275G>A	p.Arg92Gln	P	11
TNNT2	c.280C>T	p.Arg94Cys	P	1
TNNT2	c.281G>A	p.Arg94His	LP	1

TNNT2	c.304C>T	p.Arg102Trp	P	1
TNNT2	c.330T>G	p.Phe110Leu	LP	6
TNNT2	c.388C>T	p.Arg130Cys	P	4
TNNT2	c.421del	p.Arg141fs	P	1
TNNT2	c.487_489del	p.Glu163del	LP	1
TNNT2	c.487_489del	p.Glu163del	P	10
TNNT2	c.517_519del	p.Glu173del	LP	2
TNNT2	c.536C>T	p.Ser179Phe	P	1
TNNT2	c.583G>A	p.Glu195Lys	P	1
TNNT2	c.653T>C	p.Ile218Thr	LP	1
TNNT2	c.803A>T	p.Asn268Ile	LP	1
TNNT2	c.807C>A	p.Asn269Lys	LP	1
TNNT2	c.814C>T	p.Gln272*	LP	1
TNNT2	c.833G>C	p.Arg278Pro	P	2
TNNT2	c.853C>T	p.Arg285Cys	P	10
TNNT2	c.856C>T	p.Arg286Cys	LP	1
TNNT2	c.856C>T	p.Arg286Cys	P	7
TNNT2	c.860G>A	p.Trp287*	P	8
TNNT2	c.877C>T	p.Arg293Cys	LP	1
TNNT2	c.881G>A	p.Trp294*	LP	2
TPM1	c.115-1G>C		P	1
TPM1	c.184G>C	p.Glu62Gln	P	4
TPM1	c.428T>G	p.Ile143Ser	LP	1
TPM1	c.457C>G	p.His153Asp	LP	2
TPM1	c.523G>A	p.Asp175Asn	P	3
TPM1	c.548C>T	p.Ala183Val	LP	2
TPM1	c.574G>A	p.Glu192Lys	LP	1
TPM1	c.574G>A	p.Glu192Lys	P	5
TPM1	c.62G>T	p.Arg21Leu	LP	1
TPM1	c.637A>C		P	1
TPM1	c.64G>A	p.Ala22Thr	P	2
TPM1	c.655G>A	p.Asp219Asn	LP	1
TPM1	c.73G>A	p.Ala25Thr	LP	1
TPM1	c.842T>C	p.Met281Thr	LP	1
TPM1	c.842T>C	p.Met281Thr	P	2

Supplemental Table 2: Cumulative Incidence of Overall Composite Endpoint, Heart Failure, Ventricular Arrhythmias, and Atrial Fibrillation in Childhood-Onset HCM

	**KM Rates (95% CI) at Varying Follow Up Intervals (yrs)**						
	**0**	**1**	**5**	**10**	**15**	**20**	**25**
**Overall Composite**	2.6% (1.9, 3.6)	5.3% (4.2, 6.7)	10.6% (8.8, 12.6)	18.6% (16.0, 21.7)	29.6% (25.9, 33.7)	40.7% (36.1, 45.7)	48.9% (43.7, 54.4)
**Heart Failure**	0.5% (0.002,1.0)	1.3% (0.77,2.1)	3.1% (2.2, 4.5)	6.6% (5.0, 8.7)	10.3% (8.0, 13.3)	16.6% (13.2, 20.8)	21.9% (0.18, 0.27)
**Ventricular Arrhythmia**	3.4% (2.5, 4.5)	4.6% (3.6, 5.9)	6.2% (4.9, 7.7)	8.8% (7.1, 10.9)	12.3% (9.9, 15.2)	14.7% (11.8, 18.2)	16.9% (13.5, 21.0)
**Atrial Fibrillation**	1.1% (0.65, 1.9)	1.2% (0.72, 2.0)	2.8% (1.9, 4.1)	5.9% (4.3, 8.0)	11.7% (9.1, 15.1)	19.8% (15.9, 24.5)	26.8% (21.8, 32.5)

Supplemental Figure 1: Clinical Outcomes in Infant-Onset (<1 yo) vs Childhood-Onset (1-18 yo) HCM

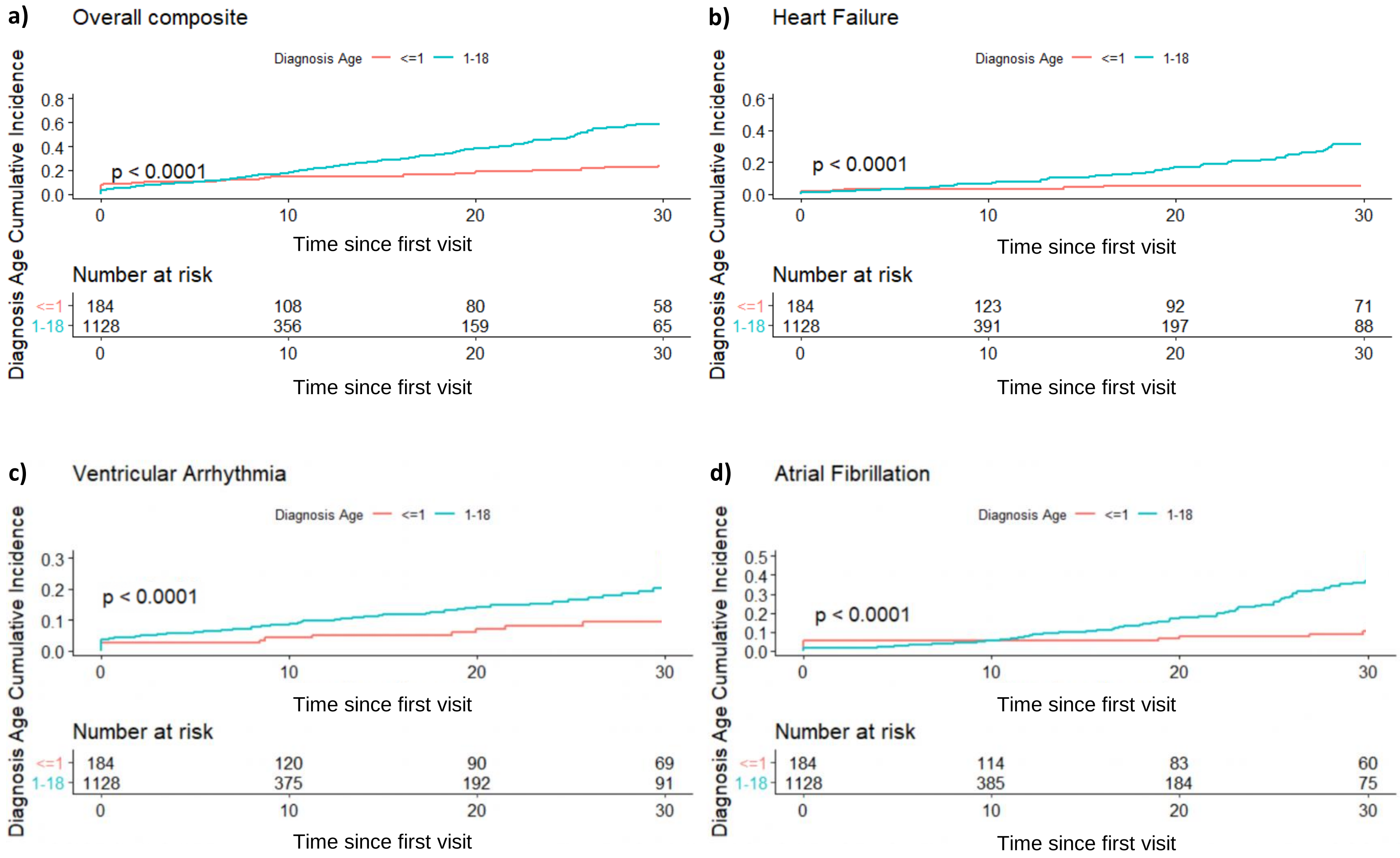

Supplemental Figure 2: Clinical Outcomes by Sarcomere Status in Childhood-Onset HCM (1-18 yo)

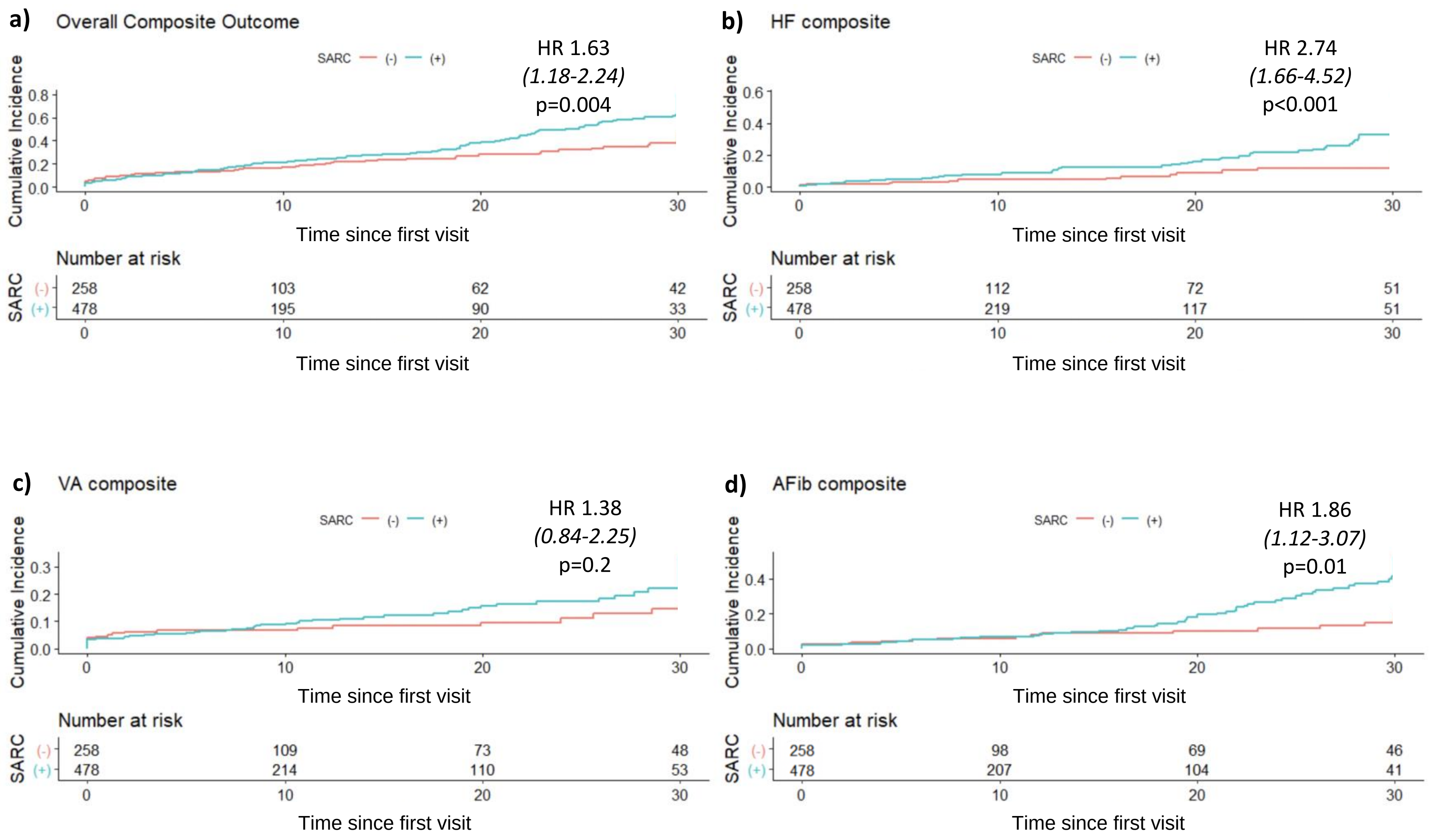

Supplemental Figure 3: Composite Outcome by Sex in Childhood-Onset HCM (1-18 yo)

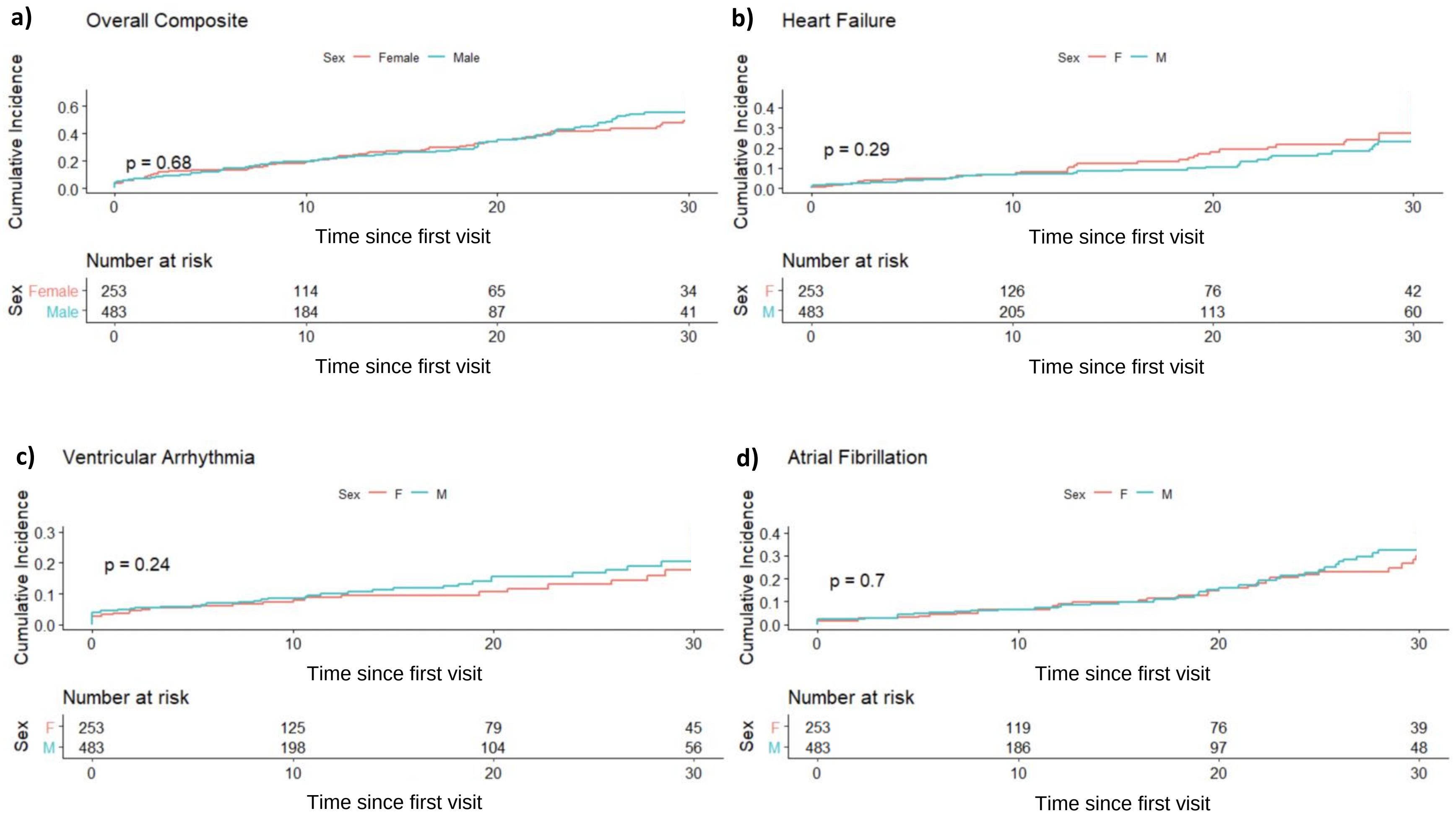

Supplemental Figure 4: Comparison of Outcomes in Childhood-Onset (n=617) vs. Adult-Onset HCM stratified by age (a) 19-39 (n=1250) and (b) >=40 (n=2529)

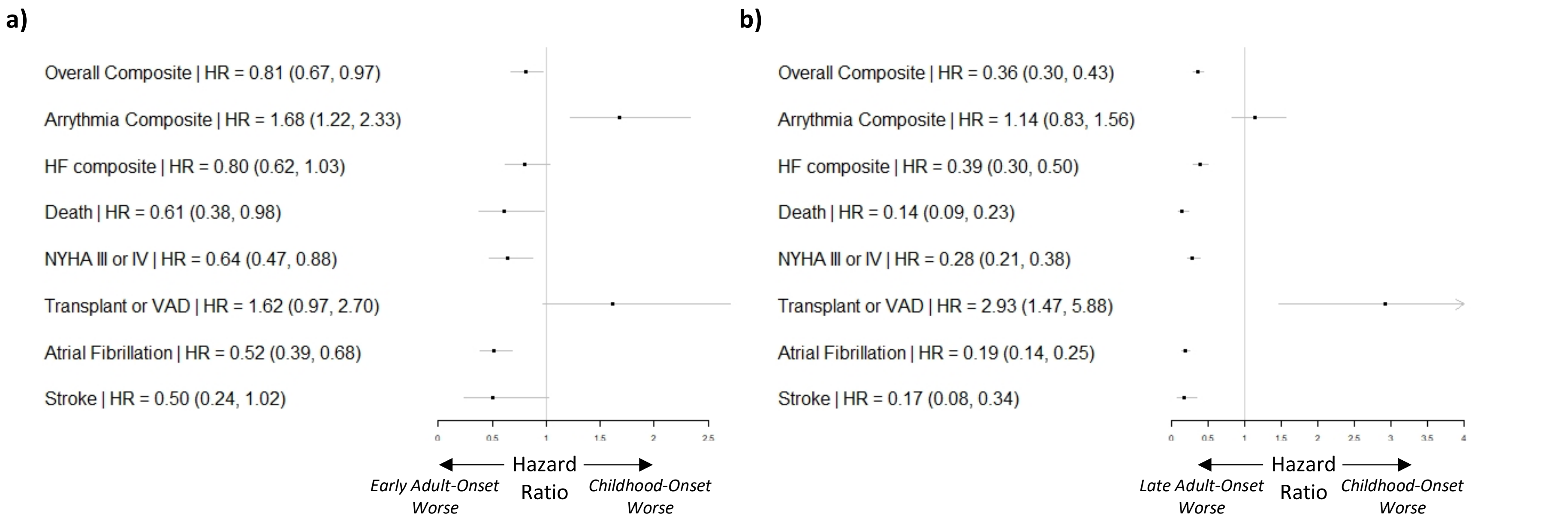

Adjusted for sex, proband status, sarcomere status, and age at diagnosis

Supplemental Figure 5: Comparison of Outcomes in Childhood-Onset Sarcomeric (n=382) vs. non-Sarcomeric HCM (n=151)

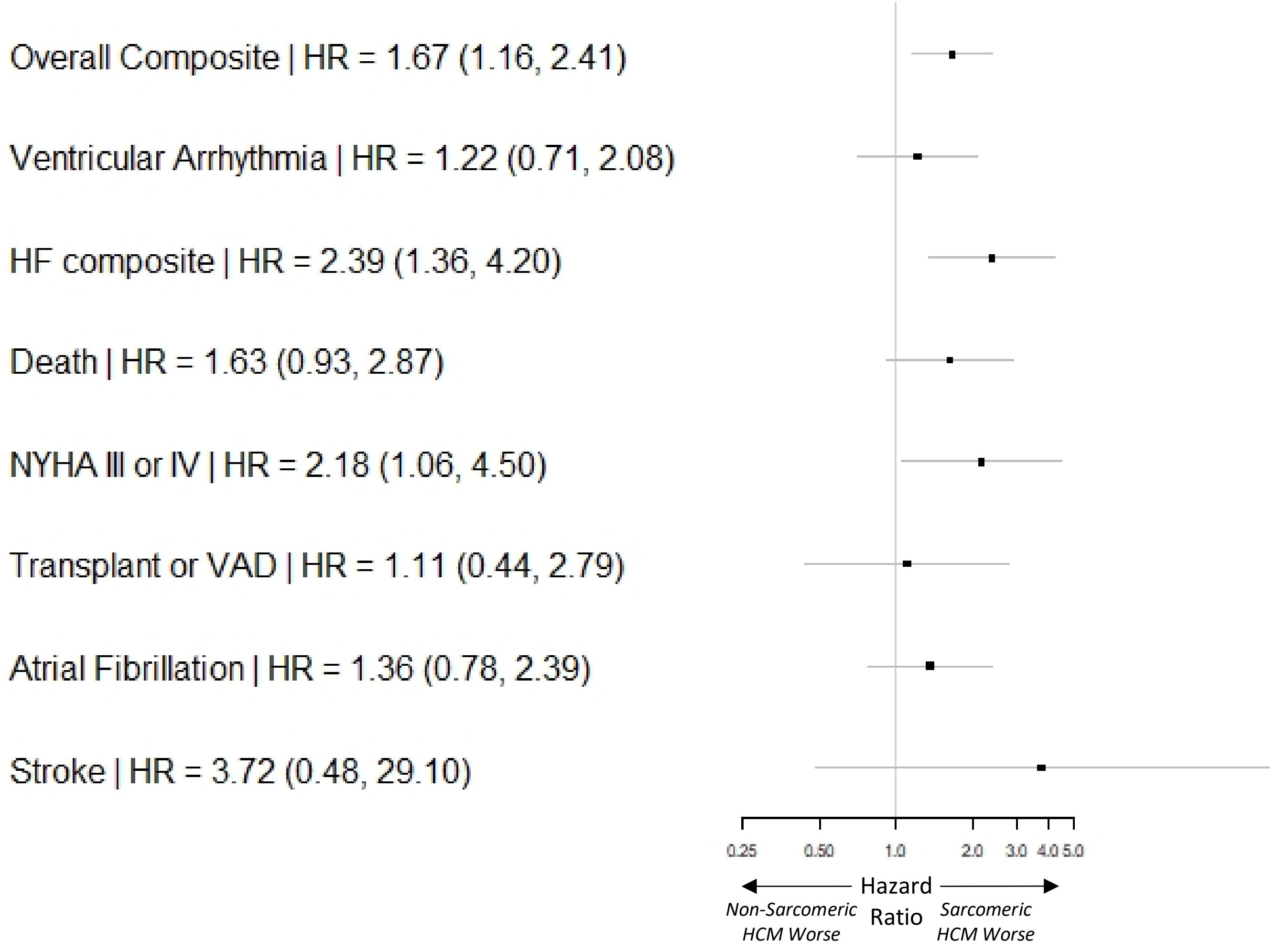

Adjusted for sex, proband status, and age at diagnosis

Supplemental Figure 6: Comparison of Outcomes in Adult-Onset Sarcomeric (n=1992) vs. non-Sarcomeric HCM (n=1787)

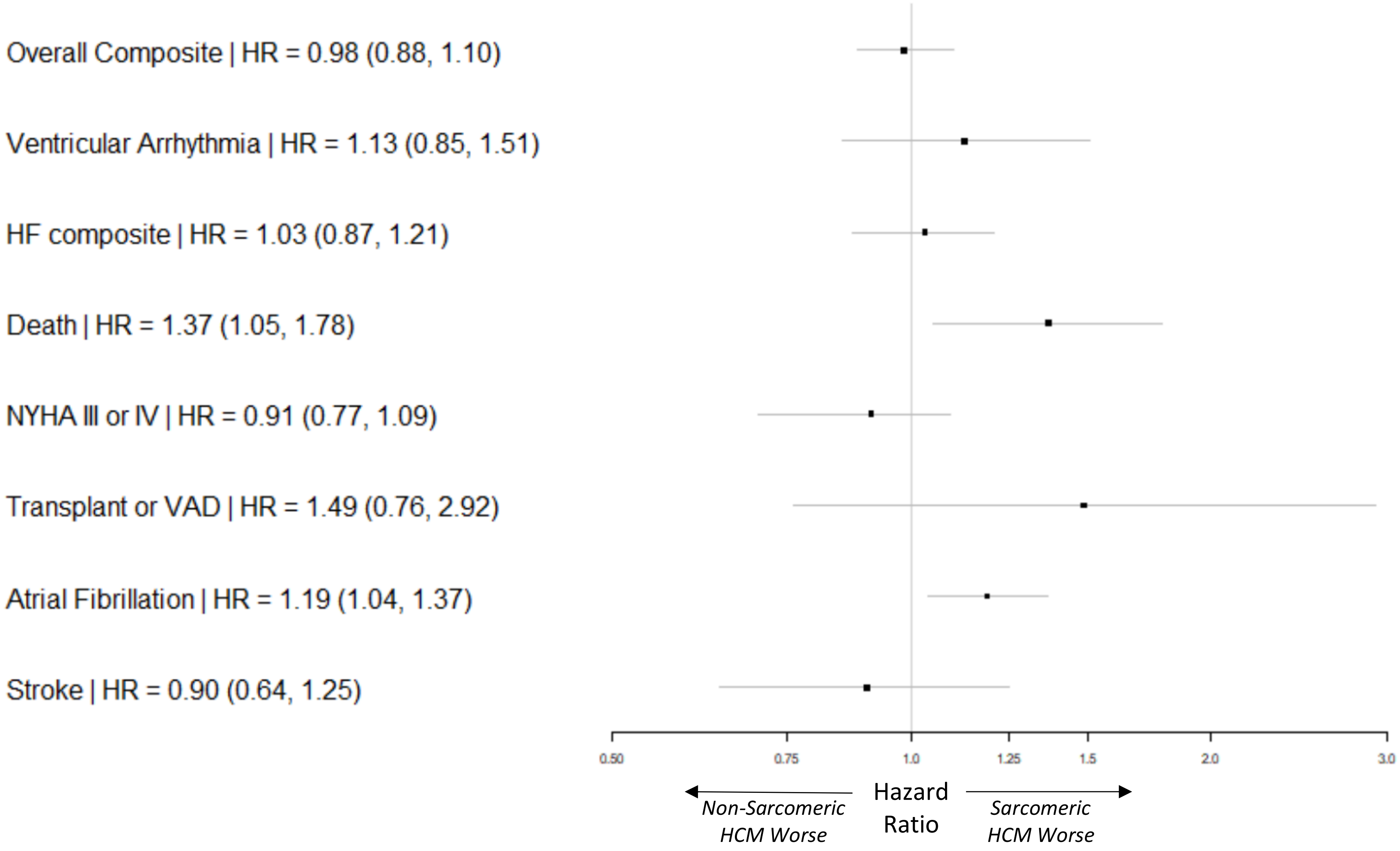

Adjusted for sex, proband status, and age at diagnosis

Supplemental Figure 7: Analysis of Outcomes by Age of Diagnosis as a Continuous Variable

We examined potentially nonlinear relationships between continuous age and the log hazard ratio of each outcome, along with corresponding 95% confidence intervals (smoothHR package in R). Each of these models is adjusted for sex, proband status, and sarcomere status. The results are consistent with Supplemental Figure 4, with childhood-onset HCM being associated with increased risk of the Ventricular Arrhythmia composite and transplant/VAD, and lower risk of heart failure, atrial fibrillation, and the overall composite.

a) Overall composite:

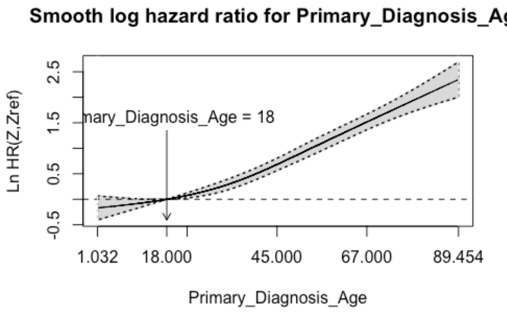

b) VA composite:

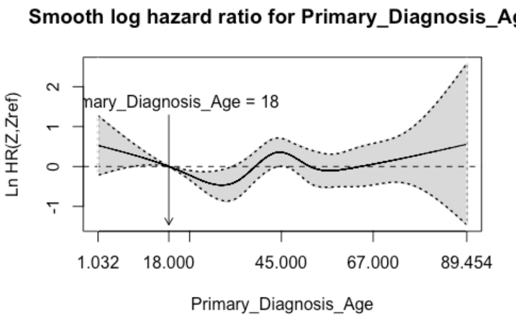

c) HF composite:

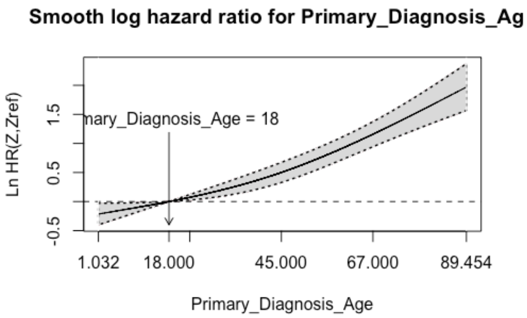

d) Transplant/VAD:

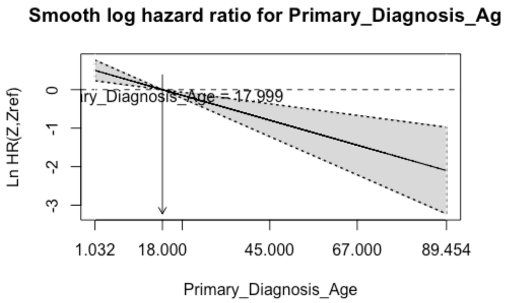

e) AFib:

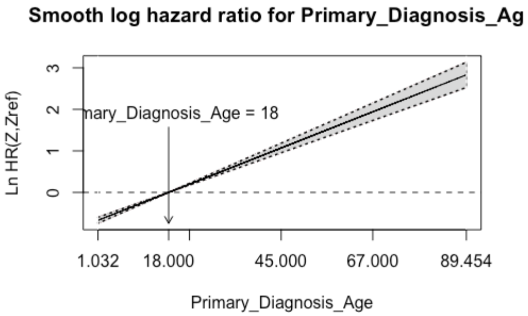

Supplement: ehab148_Supplementary_Appendix [file ehab148_supplementary_appendix.pdf]
